# Auto-sumoylation of UBC9 coordinates meiotic prophase and protects ovarian reserves

**DOI:** 10.64898/2026.08.04.742908

**Published:** 2026-08-08

**Authors:** Sunkyung Lee, Tianhong Lu, Dhananjaya S Kulkarni, Delin Zhang, Britney Ly, Sida Li, Megan Sarringhaus, Yungdae Ryu, Neil Hunter

## Abstract

The small ubiquitin-like modifier SUMO regulates key events of meiosis, including pairing and crossing over between homologous chromosomes. Auto-sumoylation of the SUMO E2-conjugating enzyme UBC9 at lysine 14 alters its substrate selectivity in vitro, but the role of this modification in vivo is unknown. Here, we show that UBC9-K14 auto-sumoylation helps coordinate meiotic prophase and is important for maintenance of the ovarian reserve. Ubc9K14R/K14R knock-in mice show a variety of defects in meiotic prophase I, including altered assembly of DNA strand-exchange complexes, delayed and defective homolog synapsis, and unstable crossover recombination complexes. In spermatocytes, these defects are associated with reduced efficiency of crossing over between the X and Y chromosomes. Oocytes from Ubc9K14R/K14R females show related but distinct defects in recombination and synapsis. Moreover, maintenance of the primordial follicle reserve is defective in Ubc9K14R/K14R females, and their fecundity is reduced. Finally, we identify the meiosis-specific homolog-axis protein SYCP3 as a direct target of UBC9 in vitro and show that SYCP3 modification is strongly stimulated by K14 auto-sumoylation. We infer that auto-sumoylation enables UBC9 to modify a subset of targets in vivo, helping to coordinate key events of meiotic prophase and enhance the survival of primordial follicles to maximize fecundity.

## Full Text Availability

The license terms selected by the author(s) for this preprint version do not permit archiving in PMC. The full text is available from the preprint server.

